# Supplementary material for: Analysis of PPARγ Signaling Activity in Psoriasis
Source: Int J Mol Sci. 2021 Aug 10;22(16):8603. doi: 10.3390/ijms22168603 (PMC8395241; doi:10.3390/ijms22168603)
Supplement: Supplementary file 1 [file ijms-22-08603-s001.zip › Supplemental materials_Analysis of PPARg signaling activity in psoriasis/Pathway models/Models images and html files/PPARG expressed in cells assotiated with psoriasis/225568.html]

stem cell


# CellType stem cell

|  |  |
| --- | --- |
| URN | urn:agi-ncimcelltype:C0038250 |
| Total Entities | 105 |
| Connectivity | 17514 |
| Name | stem cell |

---

|  |  |
| --- | --- |
| ChildConcepts | stem cell differentiation |
|  | stem cell development |
|  | stem cell renewal |
|  | stem cell proliferation |
|  | lipoblast |
|  | spongioblast |
|  | epithelial stem cell |
|  | neoplastic stem cell |
|  | myoblast |
|  | epidermal stem cell |
|  | amniotic fluid stem cell |
|  | embryonic stem cell |
|  | pluripotent stem cell |
|  | dental pulp stem cell |
|  | germline stem cell |
|  | cardiac stem cell |
|  | intestinal stem cell |
|  | muscle stem cell |
|  | hepatic progenitor cell |
|  | adult stem cell |
|  | retinal progenitor |
|  | totipotent stem cell |
|  | multipotent stem cell |
|  | oval cell |
|  | small hepatocyte |
|  | thymic epithelial stem cell |
|  | pre-thymocyte |
|  | neuroepithelial cell |
|  | hepatic stem cell |
|  | umbilical cord blood stem cell |
|  | embryoid body |
|  | fetal stem cell |
|  | proadipocyte |
|  | oligodendrocyte precursor cell |
|  | glial precursor cell |
|  | stem cell apoptosis |
|  | stem cell homeostasis |
|  | stem cell formation |
|  | stem cell migration |
|  | stem cell function |
|  | stem cell phenotype |
|  | stem cell fate |
|  | stem cell growth |
|  | stem cell survival |
|  | stem cell adhesion |
|  | stem cell viability |
|  | stem cell population |
|  | stem cell count |
|  | stem cell interaction |
|  | stem cell fate commitment |
|  | stem cell damage |
|  | stem cell regeneration |
|  | stem cell division |
|  | stem cell homing |
|  | stem cell chemotaxis |
|  | stem cell senescence |
|  | stem cell aggregation |
|  | stem cell polarity |
|  | stem cell invasion |
|  | stem cell autophagy |
|  | stem cell transendothelial migration |
|  | stem cell death |
|  | stem cell motility |
|  | stem cell transdifferentiation |
|  | stem cell sequestration |
|  | stem cell communication |
|  | stem cell disruption |
|  | stem cell localization |
|  | stem cell ratio |
|  | stem cell fate specification |
|  | stem cell elongation |
|  | stem cell size |
|  | stem cell motion |
|  | stem cell spreading |
|  | stem cell anchorage |
|  | stem cell destruction |
|  | stem cell rolling |
|  | stem cell priming |
|  | stem cell proliferative response |
|  | stem cell contact |
|  | stem cell selection |
|  | stem cell morphogenesis |
|  | stem cell cytotoxicity |
|  | stem cell fate determination |
|  | stem cell infiltration |
|  | stem cell diapedesis |
|  | stem cell phagocytosis |
|  | stem cell enlargement |
|  | stem cell lifespan |
|  | stem cell distribution |
|  | stem cell branching |
|  | stem cell dedifferentiation |
|  | stem cell anoikis |
|  | stem cell stress |
|  | stem cell structure |
|  | stem cell mediated cytotoxicity |
|  | stem cell repertoire |
|  | stem cell developmental process |
|  | stem cell replicative senescence |
|  | stem cell architecture |
|  | stem cell recognition |
|  | stem cell compartmentalization |
|  | stem cell extravasation |
|  | stem cell macrophage differentiation |
|  | stem cell tethering |

---

|  |  |
| --- | --- |
| Pathway | Elevated Receptors -> Expression Targets in Placenta |
|  | PPARG expressed in cells assotiated with psoriasis |
|  | Neighbors of stem cell |

---

|  |  |
| --- | --- |
| MedScan ID | 10004118 |

---

|  |  |
| --- | --- |
| Alias | stem cell |
|  | precursor cells |
|  | precursor cell |
|  | progenitor cells |
|  | stem cells |
|  | progenitor cell |

---

|  |  |
| --- | --- |
| NCIm ID | C0038250 |

---
